# Co-Occurrence of Hotspot Point Mutation and Novel Deletion Mutation of *TERT* Promoter in Solid Variant Papillary Thyroid Carcinoma in a Patient with Synchronous Esophageal Cancer

**DOI:** 10.3390/diagnostics11010004

**Published:** 2020-12-22

**Authors:** Jiheun Han, Young Lyun Oh, Jung-Sun Kim

**Affiliations:** Department of Pathology and Translational Genomics, Samsung Medical Center, Sungkyunkwan University School of Medicine, Seoul 06351, Korea; mint750@naver.com (J.H.); yl.oh@samsung.com (Y.L.O.)

**Keywords:** TERT, promoter, thyroid, papillary carcinoma, solid

## Abstract

(1) Introduction: Telomerase reverse transcriptase (TERT) promoter mutations are associated with unfavorable clinical outcomes in papillary thyroid carcinomas (PTCs). Two substitution mutations, C228T (c.1-124C>T) and C250T (c.1-146C>T), make up most of the mutations and occur in a mutually exclusive manner. (2) Case presentation: A 72-year-old man was initially referred to a tertiary hospital for treatment of esophageal cancer. Preoperative imaging revealed a 3.2 cm thyroid nodule pathologically diagnosed as PTC on needle biopsy. The patient underwent thyroid lobectomy with esophagectomy and was finally diagnosed with synchronous solid variant PTC (SVPTC) and esophageal squamous cell carcinoma. Sanger sequencing using DNA from the thyroid tumor showed an indel mutation, c.1-132_1-124delinsT, composed of a deletion (c.1-132_1-125del) as well as a hotspot mutation (c.1-124C>T(C228T)) in the TERT promoter. (3) Conclusions: This is the first report of PTC harboring a novel deletion along with a hotspot mutation in the TERT promoter in a patient with synchronous esophageal squamous cell carcinoma.

## 1. Introduction

Papillary thyroid carcinoma (PTC) is the most common histologic type of thyroid cancer, and overall indolent tumor, with a cancer-specific mortality rate less than 5% [1]. However, the prognosis is significantly affected by disease stage (which is defined by patient age at diagnosis), tumor size, extrathyroidal extension, and distant metastases. In an effort to finding a biomarker that indicates aggressive biological behavior of the tumor, telomerase reverse transcriptase (*TERT*) promoter mutations have been suggested as a useful prognostic indicator of clinical aggressiveness and poor outcome in differentiated thyroid carcinomas [2,3].

*TERT* encodes the catalytic protein subunit of telomerase, which is crucial for maintaining telomeric length and is reactivated in many cancers [4]. Telomerase activity is determined by TERT expression, which is predominantly controlled at transcription levels, and the TERT promoter is considered to be the most important regulatory element [5]. Two of the most recurrent non-coding mutations, C228T (c.1-124G>A) and C250T (c.1-146G>A), create a de novo binding site (GGAA) for the E26 transformation-specific (ETS) family of transcription factors, specifically the multimeric GA-binding protein (GABP) in the TERT promoter [6].

These two TERT promoter mutations occur frequently in poorly differentiated thyroid carcinoma (43.2%) and anaplastic thyroid carcinoma [7]. Among three major variants of PTC, the tall cell variant shows highest prevalence of TERT promoter mutation, followed by conventional and follicular variants [7].

Solid variant PTC (SVPTC) is rare, comprising about 3% of the total PTC [8]. It has a less favorable prognosis than the classic papillary type; however, it is much better than for poorly differentiated carcinoma [8]. According to previous studies, TERT promoter mutation occurs in about 12% of SVPTC [9,10].

Here, we first report the co-occurrence of a novel deletion and hotspot mutation in the TERT promoter in solid variant PTC found in a patient with synchronous primary esophageal cancer.

## 2. Case Presentation

**Figure 1:**
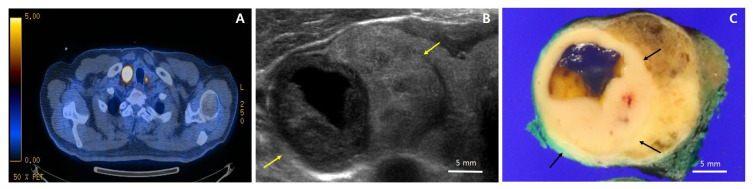
^18^F-fluorodeosy-glucose positron tomography (FDG-PET), ultrasound, and gross photos. (**A**) FDG-PET showing a hypermetabolic mass (SUVmax = 76.5) in the right thyroid as well as a focal hypermetabolic lesion (SUVmas = 7.7) in the left thyroid; (**B**) Ultrasound of the right lobe showing a 3.4 cm isoechoic solid nodule with regular margins (yellow arrows), accompanied by a 2.1 cm hypoechoic solid nodule with cystic changes and “nodule in nodule” pattern. In contrast to the larger nodule, the smaller hypoechoic solid nodule is taller than it is wide; (**C**) Gross photo showing a tumor with the largest diameter of 3.2 cm circumscribed by a thin capsule; a distinctive 1.5 × 1.4 cm light yellowish to white homogeneous solid portion (black arrows) with partial cystic changes and smooth borders within a tan to brown mass.

A 72-year-old man with dysphagia and pain behind the sternum was referred to a tertiary hospital for treatment of biopsy-proven squamous cell carcinoma of the esophagus. The patient had a 13-year medical history of diabetes, which was controlled, and a 30-year history of smoking a pack of cigarettes per day. He denied any remarkable family history or radiation exposure. Preoperative whole-body ^18^F-fluorodeosy-glucose positron tomography (PET/CT ^18^F-FDG) examination did not show any demonstrable abnormal FDG uptake in the esophagus. However, it revealed a hypermetabolic mass (SUVmax = 76.5) in the right thyroid as well as a focal hypermetabolic lesion (SUVmas = 7.7) in the left thyroid (Figure 1A). Ultrasound examination found 3.4 cm and 1.4 cm isoechoic nodules in the right and left thyroid lobes, respectively. The right lobe mass was distinctively accompanied by a 2.1 cm hypoechoic nodule with cystic changes and “nodule in nodule” appearance, and it was interpreted as an intermediate suspicious nodule (Figure 1B). The nodule of the left lobe was interpreted as a low suspicious nodule. To rule out the metastasis of esophageal cancer to thyroid, needle biopsy was performed on the right thyroid mass, which was diagnosed as papillary thyroid carcinoma. The patient underwent right thyroid lobectomy with central neck dissection for thyroid mass and McKeown esophagectomy with two-field lymph node dissection for esophageal cancer.

The Institutional review board of the Samsung Medical Center has granted permission for this case report to be published on condition that no patient-identifiable data (including patient name and photograph) are included. The approval was obtained 15 September 2020 under the following number: SMC 2020-09-025. Informed written consent for publication was not asked for by the Institutional review board, since no data that can potentially and clearly identify the patient were found in the case description.

**Figure 2:**
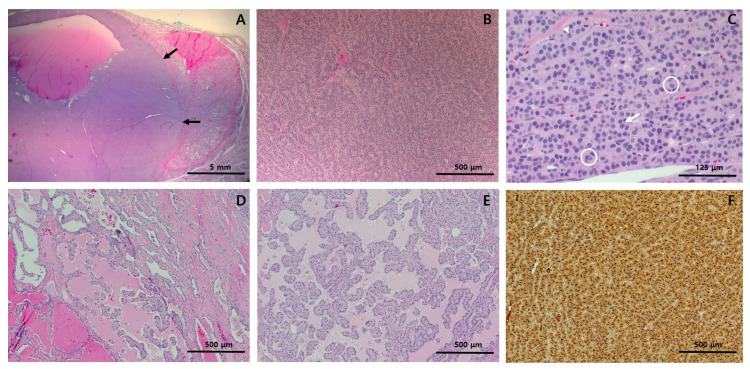
Histological features and immunohistochemistry results of the right thyroid lobectomy specimen. (**A**) At low-power view, the solid portion (black arrows) constitutes more than half of the tumor; (**B**) At medium-power view, the solid portion showed trabecular and insular growth patterns; (**C**) At high-power view, the solid portion was found to be predominantly composed of uniform cells with abundant pale eosinophilic cytoplasm. They also had nuclear features characteristic of papillary carcinoma, including pseudoinclusions (circle) nuclear grooves (arrow), and irregular nuclear contour (arrow head). (**D**,**E**) The tumor area surrounding the solid portion showed highly irregular follicles, many of which have papillary infoldings and colloid scalloping. The tumor cells showed typical papillary carcinoma nuclear features. (**F**) Immunohistochemically, the tumor cells were positive for thyroglobulin, indicating thyroid origin. Staining method: (**A**–**E**), hematoxylin and eosin; (F), polymer method. Original magnification: (**A**), ×10; (**B**), ×100, (**C**), ×400; (**D**), ×100; (**E**), ×100; (**F**), ×100.

The thyroid gross mass measured 3.2 × 2.5 cm in the largest dimension and was circumscribed by a thin capsule. On serial section, a distinctive 1.5 × 1.4 cm light yellowish to white homogeneous solid portion with partial cystic changes and smooth borders was found within the tan to brown mass (Figure 1C). Histologically, the homogeneous solid portion showed trabecular and insular growth patterns (Figure 2B), which were predominantly composed of uniform cells with abundant pale eosinophilic cytoplasm (Figure 2C). Mitoses were occasional (1–2/10HPFs), and tumor necrosis was not found. The tumor area surrounding the solid portion showed highly irregular follicles with papillary infoldings and colloid scalloping, and tumor cells revealed a polygonal shape with abundant cytoplasm (Figure 2D,F). In the whole areas of the tumor, the neoplastic cells had the nuclear features characteristic of papillary carcinoma, including irregular nuclear contours, nuclear clearing, grooves, and pseudoinclusions (Figure 2C,E). Immunohistochemically, the tumor cells were positive for thyroglobulin (mouse monoclonal, clone 2H11+6E1, 1:400, Sigma-Aldrich, Rocklin, CA, USA), indicating thyroid origin (Figure 2F). As the tumor had more than 50% solid architecture (Figure 1A), it was finally diagnosed as solid variant PTC (SVPTC). Extrathyroidal extension, blood vessel invasion, and regional lymph node metastasis of the tumor were not identified. The esophagectomy specimen revealed a 2.5 × 1.0 cm ulcerative lesion in the lower third of the esophagus. Microscopically, the tumor was characterized as moderately differentiated squamous cell carcinoma extending to submucosa. The tumor metastasized to three regional lymph nodes.

**Figure 3:**
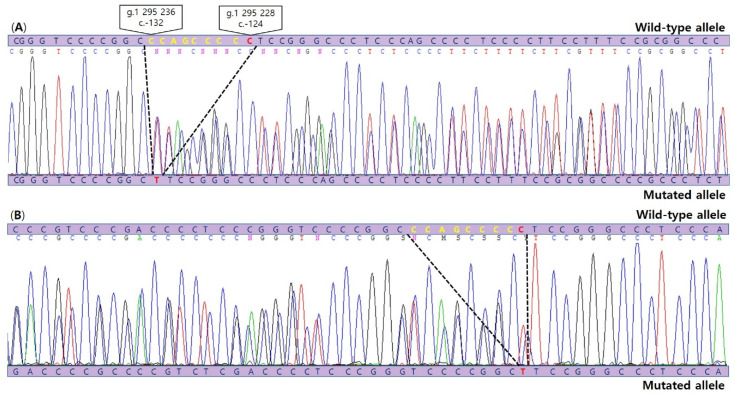
Sanger sequencing chromatograms (**A**: forward sequence, **B**: reverse complement sequence) illustrating detection of the c.1-132_1-124delinsT, composed of a novel 8 bp deletion mutation (c.-132_c.-125del) (yellow) as well as the common C228T(c.1-124C>T) TERT promoter mutation (red) in SVPTC.

DNA from formalin-fixed, paraffin-embedded tissue from SVPTC was extracted. We carried out Sanger sequencing to identify *TERT* promoter mutations using a set of primers 5′-AGTGGATTCGCGGGCACAGA-3′ (forward) and 5′-AGCACCTCGCGGTAGTGG-3′ (reverse). The analysis revealed a novel indel mutation, c.1-132_1-124delinsT, composed of a deletion (c.1-132_c.1-125) along with the well-known substitution mutation (c.1-124(G>A)(C228T)) (Figure 3).

No evidence of recurrence was observed during seven months after the lobectomy. Complete thyroidectomy for the nodule of left lobe was performed afterwards, and it was finally diagnosed as follicular adenoma.

## 3. Discussion

TERT promoter mutations are identified in various types of cancers with different frequencies [11]. In thyroid cancer, TERT promoter mutations are more frequently found in poorly differentiated thyroid carcinoma and anaplastic thyroid carcinoma than PTCs [12]. As a result of its rarity, the frequency of TERT promoter mutations in SVPTC has not been well known, and only a few studies compared the genetic alterations among subvariants of PTC including SVPTC. According to these studies, the prevalence of TERT promoter mutations in SVPTC is slightly higher than that in conventional PTC (cPTC) but much lower than that of tall cell variant PTC [9,10].

Previous studies demonstrated that TERT promoter mutations in differentiated thyroid carcinomas was associated with some clinicopathological features, including older age at diagnosis, larger tumors, higher frequency of distant metastases, and worse tumor stage [2]. In our case, the older patient age (72 years old) and larger tumor size (3.2 cm) are relevant to the presence of the TERT promoter mutation. Although the clinical implication of this subtype is undetermined, a recent systemic review suggested that SVPTC should be regarded as an aggressive variant of PTC because of its higher propensity for vascular invasion and tumor recurrence [13]. Thus, a more aggressive approach, including total thyroidectomy accompanied by lymph node dissection, may be required.

Two mutations of the TERT core promoter, which cause a cytidine-to-thymidine (CT) dipyrimidine transition at chromosome 5 1,295,228 and 1,295,250 (−124 and −146bp from the ATG), c.1-124C>T(C228T) and c.1-146C>T(C250T), respectively, predominantly occur regardless of tumor type [14,15]. The C228T mutation is more prevalent than C250T among various malignancies including thyroid cancer [16,17]. The mutations occur in a mutually exclusive manner, suggesting their functional redundancy. Multiple studies provide evidence that the presence of C228T or C250T mutations promotes telomerase activity both in vivo and in vitro [6,18,19]. These mutations generate a de novo binding site for activating the ETS family of transcription factors, including GA-binding proteins (GABP), thereby activating TERT transcription and telomerase activity [6,20].

**Figure 4:**
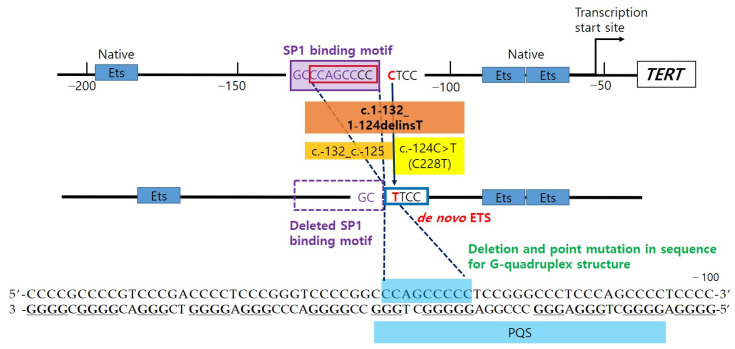
c.1-124C>T creates de novo E26 transformation-specific (ETS), which cooperates with native ETS, to recruit activating transcription factor (GA-binding protein, GABP). c.1-132_1-125 deletion (8bp, 1.3 helix turn) eliminates the transcription factor SP1 binding site, which is identified using TFBIND (http://tfnbind.hgc.jp/), and it also closes the distance between native ETS and de novo ETS (C228T). In addition, the mutations are located in the putative quadruplex sequence (PQS) that are able to fold in G-quadruplex structure.

Although not a promoter lesion, one previous study reviewing 173 Hürthle cell carcinomas of thyroid cases found a 24 bp deletion in the 5′ UTR of exon 1 (c.-53_-29delAGCGCTGCGTCCTGCTGCGCACGT) of TERT in one sample [21]. A recent study identified two additional novel mutations in the thyroid tumor: c.-332C>T in one medullary thyroid carcinoma and c.-104_-83dup in one PTC [22]. They suggested these mutations might create additional ETS-binding sites. Our novel 8 bp deletion was found just upstream of the C228T mutation, and it does not seem to generate an additional transcriptional factor binding motif. Instead, the mutation deletes the Specificity Protein 1 (SP1) binding site (Figure 4). A previous study investigated the putative G-quadruplex-forming sequence (PQS) embracing 3 Sp1 binding sites, between −90 and −22 upstream of the transcription start site of TERT promoter [23]. The study suggested that the formation of a tandem G-quadruplex structure might produce a significant inhibitory effect by masking Sp1 binding sites, and it also confirmed the importance of loop size to G-quadruplex stability reported in several studies [24]. The mutated sequence in our case is included in one of the PQS, which is able to form stable G-quadruplex found between the −167 and −100 region of the TERT promoter [25] (Figure 4). The co-occurrence of deletion and point mutation in the PQS may have an effect on the stability of the G-quadruplex structure. The deletion mutation also affects spacing between pre-existing and de novo regulatory elements. In a previous study, the authors observed that hotspot mutant promoter activity fluctuated depending on the distance from the native ETS sites, and there was peak activity when two locations were separated by full helical turns of DNA, suggesting that TERT promoter mutation cooperates with native ETSs to recruit GABP [6]. In order to confirm the overall effects of deletion and point mutation of the TERT gene promoter, further studies should be performed through luciferase reporter assay.

The co-occurrence of different substitution mutations of the TERT promoter has previously been reported. One previous PTC cohort study reported that mutations C229T (c.-125C>T) and C232T (c.-128C>T) were simultaneously found in one PTC sample [10]. Similarly, SNP and somatic hotspot mutations can co-exist in TERT promoter lesions. The rs2853669 common allele, a common T>C polymorphism at the −245 bp position from the ATG site, disrupts the pre-existing non-canonical ETS2 binding site in the proximal region of the TERT promoter, reducing the transcriptional activity of TERT [26]. Several studies reported that the prognostic effect of TERT promoter mutations was modified by the SNP in the TERT promoter. In the presence of a somatic TERT promoter mutation in the tumor, patients with the rs2853669 common allele showed poor prognosis in bladder cancer [27] and glioma [28], although conflicting evidence has been found for the latter [29]. However, the co-occurrence of different types of TERT mutations or variations has never been reported thus far.

A previous study evaluating two hotspot TERT promoter mutations in 313 esophageal squamous cell carcinoma samples identified 1.6% occurrence, showing the extremely low frequency compared to that of thyroid cancer (15–50%) [30]. Some studies identified a germline mutation in TERT, c.-57T>G in only two melanoma families worldwide, indicating that this inherited mutation is extremely rare [31,32]. These families were characterized by early age of melanoma onset (average 34 years) and evidence of susceptibility to other primary cancers (ovary, renal cell carcinoma, bladder, breast, and lung). Therefore, although we did not test for the TERT promoter mutation in tissue from esophageal cancer, it is unlikely that the TERT promoter mutation influences the occurrence of synchronous tumors.

The incidence of secondary cancer is 4.3% to 10.4% in patients with primary esophageal cancer [33,34,35,36]. Common secondary primary tumor sites include aerodigestive tract organs, such as the oral cavity, pharynx, larynx, lungs, and stomach [34], and the treatment and prognosis for these patients are determined primarily by the initial esophageal cancer [36]. The incidence of multiple primary cancers involving the esophagus and thyroid is low, and only less than 20 patients have been reported [37]. Most of these patients had advanced clinical staging of cancer. Histologically, esophageal cancer has been diagnosed as squamous cell carcinoma in all patients. Thyroid cancer has been evaluated as papillary carcinoma or follicular carcinoma.

In conclusion, this is the first report to describe the co-existing deletion and hotspot TERT promoter mutation of SVPTC in a patient with synchronous esophageal squamous cell carcinoma.
